# A Culturally Competent Approach to Discharge Planning and Transfer of Care

**DOI:** 10.7759/cureus.50235

**Published:** 2023-12-09

**Authors:** Norka Quillatupa, Cecilia S Covenas

**Affiliations:** 1 Geriatrics, University of California, Los Angeles (UCLA) - Kern Medical, Bakersfield, USA; 2 Family Medicine, Rio Bravo Family Medicine Program, Bakersfield, USA

**Keywords:** effective hospital discharge planning, adverse safety events, transitional care components, safe transition, cultural competency, transfer of care, discharge planning

## Abstract

Culturally competent discharge planning and transfer of care play a leading role in communication and the effective provision of high-quality care to patients from diverse sociocultural backgrounds. However, no standardization has been established.^ ^Here, we present the case of a Spanish-speaking patient discharged with instructions in English on two separate occasions, which resulted in readmission and deleterious outcomes. We emphasize the need to provide a safe and culturally competent transition of care.

## Introduction

The transition from any healthcare system to home can be challenging for patients. In this process, they receive a significant amount of crucial information, such as learning about a new diagnosis, changes or discontinuation of medications, and follow-ups with new and old specialties, among other details. On the other hand, the hospital's discharge planning and transfer of care lack standardization, making this process even more complicated. As a result, patients are exposed to various threats to safety, including providing discharge instructions in a different language, misunderstanding medication management, worsening of previous conditions, and readmissions [1].

This concern is not new, and efforts to mitigate these adverse events have been studied. The National Transitions of Care Coalition has three models aiming for a safe and seamless transition that highlight that efforts should consider social determinants of health, among other recommendations. [2]. Also, the project Achieving Patient-Centered Care and Optimized Health in Care Transitions by Evaluating the Value of Evidence (ACHIEVE) recognizes transitional care components based on learning about the specific needs of patients [3,4].

In this context, culturally competent discharge planning and transfer of care have a central role in communicating with and effectively providing high-quality care to patients from diverse sociocultural backgrounds [5]. According to a review, there is a promise for cultural competency to reduce adverse safety events; however, there is a clear need for robust studies that look specifically at associations between race and culture [6-8]. Additionally, efforts to identify factors contributing to poor transitions and improvements in discharge planning and transition of care have been made, yet no standardization has been established. Studies have encouraged care providers and policymakers to look carefully into the discharge problems in their local settings and select appropriate solutions for improving hospital discharge effectively [8].

This article was accepted and presented as a poster at the American Geriatric Society 2023 Annual Scientific Meeting held from May 3rd, 2023 to May 6th, 2023, in Long Beach, California, USA.

## Case presentation

This case involves a 74-year-old Hispanic, Spanish-speaking male patient with a complex medical history, including end-stage renal disease on hemodialysis, hypertension, gout, type 2 diabetes mellitus, benign prostate hyperplasia, venous stasis ulcers, and an ascending abdominal aneurysm. The patient is non-adherent to medical treatment, including medications, appointments, and diagnostic testing. During the patient's history-taking session in his native language, he expressed unawareness of appointments with different specialties and difficulties keeping track of them despite attempts to write them down. Moreover, he exhibited uncertainty regarding the implications and complications of newly diagnosed diseases. Additionally, the patient had a level of education comparable to that of middle school and lived alone in a mobile home. He maintained independence in all basic activities of daily living (ADLs), such as bathing and feeding, and instrumental activities of daily living (IADLs), including managing finances, medication, and shopping.

During his initial hospitalization, the patient was admitted due to medication-related misunderstandings, resulting in worsening kidney function. On the first day of hospitalization, three of his regular medications were discontinued, atrial fibrillation was diagnosed, and anticoagulation therapy was initiated. He was discharged the following day with instructions regarding new medications, changes in dosages and frequencies of previous medications, and appointments for at least two specialty follow-ups. Notably, all these discharge instructions were provided in English.

During a clinic follow-up visit one week after hospital discharge, the patient claimed to be taking "everything as prescribed," including a medication discontinued during his previous hospitalization and continuing to take old medications at the same frequency as before his initial hospitalization. Additionally, he did not recall being prescribed anticoagulants. Furthermore, the patient, previously ambulatory and fully independent in ADLs and IADLs, was now a wheelchair user due to weakness and generalized pain.

Subsequently, the patient was readmitted due to worsening kidney function, uremia, and a gout flare. New medications, such as colchicine, were added during this hospitalization, while others, including furosemide, were discontinued. Upon discharge, the patient received medication discharge instructions in English for gout treatment. One day post-discharge, a Spanish-speaking provider conducted medication reconciliation over the phone. During a follow-up appointment one week later, it was noted that the patient was adhering to the prescribed medication regimen.

## Discussion

The discharge process from the hospital represents a critical juncture in a patient's healthcare journey. Establishing standardized discharge protocols can potentially mitigate quality and safety gaps during the transition from the hospital to outpatient care [9]. However, it is crucial to recognize that merely implementing standardized discharge planning is insufficient. It is evident that culturally appropriate discharge planning and transition of care are essential to ensuring an effective process.

In this particular case report, we present the experience of an older adult patient with multiple comorbidities who, within a short period, was readmitted to the hospital due to medication-related misunderstandings. The underlying cause was attributed to culturally inappropriate discharge instructions, customized for individuals with higher levels of medical literacy and fluency in English. These instructions lacked the necessary efficiency and effectiveness in providing tailored information, resulting in a deterioration of the patient's pre-existing conditions and subsequent readmission.

A review aimed at enhancing healthcare for culturally and linguistically diverse patients identified five critical components of culturally competent healthcare facilities: (1) the presence of bilingual and bicultural medical professionals who share a similar cultural background with their patients; (2) incorporate culturally specific concepts into one-on-one interactions between patients and healthcare providers, including understanding the patient's concerns, barriers to accessing healthcare, cultural values and norms, experiences related to migration, use of culturally specific language patterns, and culturally competent communication methods; (3) use culturally and linguistically adapted written or visual materials, such as translating materials into the patient's native language, adjusting them to the patient's level of literacy and education, including culturally sensitive treatment recommendations, and addressing barriers to care; (4) involve families in the healthcare process; (5) ensure continuity of care [10].

It has been shown that the quality of discharge planning is associated with a decreased likelihood of hospital readmission. A study on improving hospital discharge planning for frail older individuals stated that it requires targeted interventions that address key areas such as family inclusion and education, effective communication between healthcare workers and family members, interdisciplinary collaboration, and sustained support after discharge [11]. Some examples of successful discharge planning are shown in a systematic review and meta-analysis that revealed a meaningful relationship between communication intervention at discharge and improved adherence to treatment, greater knowledge retention, and enhanced patient satisfaction, observed 30 days after discharge [12].

An ethnographic investigation demonstrated the importance of culturally competent discharge planning. It has been shown that older adults and their family members highly appreciate medication discharge education. This was particularly true when the information was tailored to their needs, presented verbally, and then reinforced in writing [13].

It is worth highlighting educational tools to enhance patient knowledge and reduce medication errors. The Department of Health and Human Services, the Agency for Healthcare Research and Quality, and the National Council on Patient Information and Education recommend using a Medicine Record Form. This form should include essential elements such as unit strength, indication, appearance, and cautionary information [14].

Also, the Durable Display at Discharge (3D) tool was designed to promote patients' understanding of the medication. The 3D tool had ample space to affix and display tablets or pills, including the trade name (if applicable), unit strength, the number of units to be taken, purpose (indication), comment or caution, a larger font, card stock durability, and a reconciliation feature. It brought better patient satisfaction and low rates of self-reported medication errors [15].

In summary, culturally competent discharge planning is a multi-component and complex process that must be addressed from different angles, including patients, healthcare providers, and institutions. Creating potential training programs or interventions that comprehend the cultural and linguistic barriers, disparities in health literacy, fears of stigma, mistrust in the healthcare system, personal beliefs, and how social determinants of health shape a patient's experiences are vital in promoting treatment adherence and achieving improved health outcomes.

## Conclusions

To ensure an effective and comprehensive transfer of care, particularly during the critical period of discharge from inpatient to home, it is imperative to thoroughly consider all relevant social determinants about the patient, including their cultural, ethnic, and literacy background. It is crucial to underscore the necessity for further research studies that focus on standardizing culturally appropriate discharge planning and transitioning of care. This emphasis aims to ensure a seamless and satisfactory process, considering diverse patient populations' unique needs and requirements.
